# Mental Health Informatics

**DOI:** 10.1192/pb.bp.113.043885

**Published:** 2014-10

**Authors:** Sean Maskey

**Figure:**
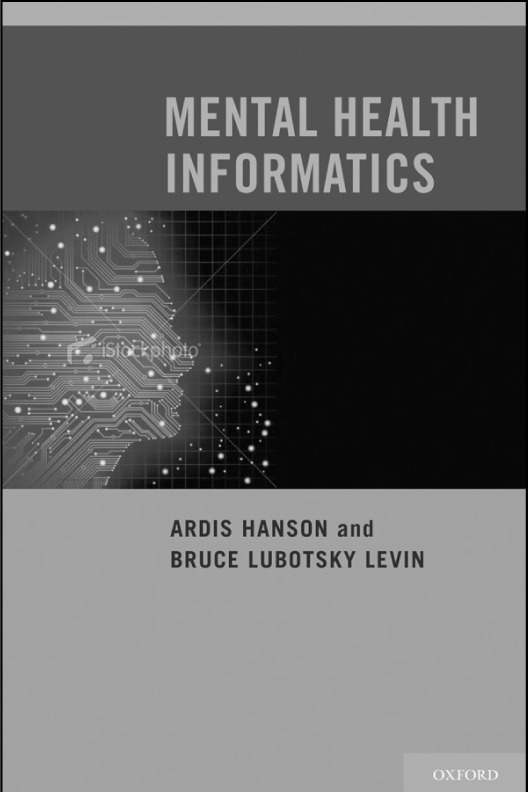


This is a small, dense book written by two American academics as a primer for their undergraduate course in (health and) mental health informatics. It is unashamedly US focused; the final section on an international perspective is devoted to technologies and processes suitable for the developing world. It will be of interest to European informatics specialists in academic settings and possibly industry, but it is not for the interested clinician or even the chief clinical information officer, unless they need familiarity and credibility in the US context.

There are four sections: mental health and informatics, standards and implementation, competencies and strategies, and a short section on globalisation and the future. The style is didactic; it gives a recent history of the American sociopolitical context of health informatics and some of the challenges in the application to mental health, ranging from systems processes, patient administration systems, with the addition of billing procedures and cross-state accreditation and licensing, to the particular challenges of mental health electronic records, which are primarily narrative rather than quantitative. However, while it links changes and developments in information processing capabilities and the federal political imperative, (created by the demographic challenge of the increasing ratio of those with chronic ill health and mental health to the economically productive population), the lack of comparative analysis of the impact of various natural experiments in legislation and informatics will frustrate the reader who seeks to do more than get to know the current US model.

The chapters addressing implementation research are interesting to the non-academic, but again describe a system and methodology rather than analysing its strengths and weaknesses compared against others. This is appropriate for the specific target audience but limits the readership and does not facilitate ready extraction of knowledge and understanding from the data.

